# Value of the log odds of positive lymph nodes for prognostic assessment of colon mucinous adenocarcinoma: Analysis and external validation

**DOI:** 10.1002/cam4.4366

**Published:** 2021-11-18

**Authors:** Huajun Cai, Tianbao Xu, Zhicheng Zhuang, Yiyi Zhang, Yuan Gao, Xing Liu, Jinfu Zhuang, Yuanfeng Yang, Guoxian Guan

**Affiliations:** ^1^ Department of Colorectal Surgery The First Affiliated Hospital of Fujian Medical University Fuzhou China; ^2^ Department of Colorectal Surgery Fujian Medical University Union Hospital Fuzhou China

**Keywords:** colon mucinous adenocarcinoma, LODDS, nomograms, prognosis, RPA stage

## Abstract

**Purpose:**

To evaluate the impact of the log odds of positive lymph nodes (LODDS) on cancer‐specific survival (CSS) in colon mucinous adenocarcinoma (MAC) patients, compared with pN stage and the lymph nodes ratio (LNR).

**Methods:**

A total of 10,182 colon MAC patients from the Surveillance, Epidemiology, and End Results database were divided into the training group. The external validation group included 153 patients from Fujian Medical University Union Hospital. The Cox regression method was used to identify prognostic risk factors. Nomograms were evaluated by Harrell's concordance index (C‐index) and calibration curves. Recursive partitioning analysis (RPA) was used to develop a novel staging system.

**Results:**

Time‐dependent receiver operating characteristic curves (ROC) to predict CSS showed the areas under the ROC curve of LODDS were always higher than pN stage and LNR. LNR and LODDS classifications can well distinguish the prognosis of patients with the same pN stage. Cox analyses indicated that age, tumor size, pT stage, pN stage, LNR, and LODDS were independent predictors of CSS (*p* < 0.05). Based on three lymph nodes classifications, we constructed three prognostic nomograms models for CSS. The C‐index of the pN, LNR, and LODDS classification nomograms were 0.746 (95% confidence interval [95% CI]: 0.736–0.756), 0.750 (95% CI: 0.740–0.760), and 0.758 (95% CI: 0.748–0.768), respectively. In external validation, we observed the C‐index of LODDS classification nomograms was 0.787 (95% CI: 0.648–0.926). RPA stage, including four stages, was constructed successfully based on pT stage and LNR or LODDS, respectively. The 3‐, 5‐, and 8‐year areas under the ROC curve of LNR‐RPA stage and LODDS‐RPA stage were superior to tumor‐node‐metastasis stage.

**Conclusion:**

LODDS to be a better prognostic factor of CSS for colon MAC patients than pN stage and LNR. A nomogram and RPA stage base on LODDS can provide accurate information for personalized cancer treatment.

## Graphical abstract

This study revealed the prognostic value of the log odds (LODDS) of positive lymph nodes in colon mucinous adenocarcinoma (MAC), a rare pathologic type of colon cancer, by comparing with the pN stage and the lymph nodes ratio (LNR). We constructed three nomograms models (based on pN stage, LNR, and LODDS, respectively) using a population‐based cohort to predict the outcomes of MAC patients, and confirmed those using an external validation cohort. Novel staging systems were developed based on LNR and LODDS, respectively, using recursive partitioning analysis.

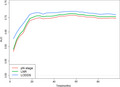

## 1 INTRODUCTION

Worldwide, colorectal cancer remains a common malignant tumor and is a dominant cause of cancer‐specific mortality.^1^, ^2^ In the context of personalized medicine, patient management based on histological types is required. Colon mucinous adenocarcinoma (MAC), a particular histological subtype of colon cancer, is characterized by mucinous components that exceed 50% of tumor tissue components.^3^, ^4^ MAC accounts for about 10%–15% of total colorectal cancer cases.^5^ At diagnosis, many MAC patients present with advanced stage and regional lymph node metastasis.^6^, ^7^, ^8^ However, the prognostic significance of MAC still is controversial. Multiple studies indicate that MAC subtype patients have a dismal survival.^9^, ^10^, ^11^ In contrast, a few studies have demonstrated that MAC does not affect survival outcomes compared to the adenocarcinoma subtype.^12^, ^13^, ^14^ Hence, given this scenario, a more suitable and accurate prognostic model is explicitly warranted for use in colon MAC patients.

Lymph node metastasis is an essential driver of clinical outcomes. The American Joint Committee on Cancer (AJCC) tumor‐node‐metastasis (TNM) classification is based on the number of positive regional lymph nodes (PLN). Nevertheless, patients with the same TNM stage remain heterogeneous. Recently, the lymph nodes ratio (LNR) and the log odds of positive lymph nodes (LODDS) are applied to clinical management and survival prediction in multiple tumors, which demonstrate distinct superiority.^15^, ^16^, ^17^, ^18^ Although some studies have reported the prognostic value of LNR and LODDS in colon patients.^19^, ^20^, ^21^ However, these studies are often directed against all histological types in colon cancer. Meanwhile, some of these lacked external validations. Therefore, the role of LNR and LODDS in MAC patients is still unclear.

In the present study, we enrolled colon MAC patients from the Surveillance, Epidemiology, and End Results (SEER) database, compared the ability of AJCC (pN stage) classification, LNR classification, and LODDS classification to predictive survival. Nomogram prognostic model was constructed base on independent prognostic factors. Furthermore, we also validated this model using patients from an external cohort.

## 2 METHOD

### 2.1 Study cohort

Colon MAC patients retrieved from the SEER database from 2004 to 2015 were assigned to the training group using SEER*Stat software (www.seer.cancer.gov/seerstat). The external validation group consisted of colon MAC patients from Fujian Medical University Union Hospital (FJMUUH) between January 2008 to September 2017. All participants meet the following inclusion and exclusion criteria. Specifically, details of inclusion criteria were: (1) pathologically confirmed MAC based on International Classification of Diseases of oncology (ICD‐O‐3); (2) underwent colon resection and regional lymph node dissection; (3) AJCC TNM stage I–III. Details of exclusion criteria were: (1) distant metastasis at the time of diagnosis or during surgery; (2) complicated with another primary tumor; (3) incomplete follow‐up data; or (4) incomplete tumor clinicopathological features data (including pT status, pN status, tumor size). Ultimately, 10,182 patients in the SEER cohort and 153 patients in the FJMUUH cohort were included. The study was approved by the Hospital Ethics Committee. Given that the SEER database is publicly accessible, informed consent in training does not require. In the external validation group, informed consent was exempted considering this retrospective design.

We retrieved clinicopathological features information from the SEER database, including age at diagnosis, sex, race, tumor site, tumor size, pT stage, pN stage, the number of total regional lymph nodes (TLN) examined, and the number of positive regional lymph nodes. Cecum, ascending colon, hepatic flexure of colon, and transverse colon were classed as proximal colon, while splenic flexure of colon, descending colon, and sigmoid colon were categorized as distal colon. And the pN status is reclassified according to the 8th edition of the AJCC staging system (pN status: pN0, pN1a, pN1b, pN2a, pN2b). Colon cancer‐specific survival (CSS) was used as the primary endpoint.

### 2.2 LNR classification

The ratio of PLN/the number of TLN was calculated as LNR. Aside from LNR = 0 and LNR = 1, the value of PLN/TLN was partitioned into 10 groups by 0.1 intervals. Kaplan–Meier (KM) analysis was performed to compare the survival differences between two neighboring groups. Then, 12 LNR subgroups were regrouped according to similar CSS.

### 2.3 LODDS classification

Log[(PLN + 0.5)/(TLN – PLN + 0.5)] was counted as LODDS. The addition of a value of 0.5 to the numerator and denominator in the formula is to avoid singularity.^22^ The classification of LODDS subgroups was similar in design to the LNR subgroups with 0.5 intervals.

### 2.4 Comparison among three lymph nodes classifications and prognostic model

A time‐dependent receiver operating characteristic (ROC) plot was performed, and the areas under the ROC curve (AUC) were counted to compare the predictive ability of three lymph nodes classifications. A univariate Cox proportional hazards model was performed to determine the prognostic values factor for CSS. The median tumor size was set as the cutoff point. The pN stage, LNR, LODDS were included in multivariate analysis, respectively. Finally, three nomogram models were constructed base on independent prognostic factors. The Harrell's concordance index (C‐index) and calibration curve were applied to evaluate the predictive ability of the three models. FJMUUH cohort was used for external validation of the nomogram model.

### 2.5 Statistical analysis

All the statistical analyses were carried out on SPSS (versions 22.0) and R software (versions 3.6.3). Difference testing between SEER cohort and FJMUUH cohort was assessed by student *t*‐test or Chi‐squared test, when appropriate. The cox regression was used for univariate or multivariate survival analyses. The “survival ROC” package and “rms” package were used to generate time‐ROC plots and nomograms, respectively. A novel tumor stage was reclassified using recursive partitioning analysis, which can be accessed from the online website (http://rpa.renlab.org).^23^ *p* < 0.05 was considered statistically significant.

## 3 RESULT

### 3.1 Clinicopathological features of patients

There were 10,182 and 153 patients in the SEER cohort and FJMUUH cohort, respectively. Table 1 illustrates two cohort baselines. Of the SEER cohort, the mean age was 68.7 (±14.5) years. Most of them (84.0%) had advanced pT stage (pT3 or pT4), and 4147 (40.7%) patients had positive lymph nodes. A majority of tumors (76.3%) were located in the proximal colon. Of the FJMUUH, the mean age was 59.0 (±13.9) years. Similar to the SEER cohort, there were 145 (94.8%) and 70 (45.8%) patients who had advanced pT stage and positive lymph nodes. The tumor size in FJMUUH cohort was larger than that in SEER cohort significantly (*p* = 0.012).

**TABLE 1:** Baseline characteristics of the SEER cohort and FJMUUH cohort

Characteristics	SEER cohort	FJMUUH cohort	*p* value
	*N* (%)	*N* (%)	
Age, years	68.7 ± 14.5	59 ± 13.9	<0.001
Sex			0.001
Male	4695 (46.1)	91 (59.5)	
Female	5487 (53.9)	62 (40.5)	
Race			<0.001
White	8400 (82.5)	0 (0.0)	
Black	1087 (10.7)	0 (0.0)	
Other/unknown	695 (6.8)	153 (100.0)	
Tumor size, cm	5.9 ± 3.6	6.3 ± 2.1	0.012
Tumor site			<0.001
Proximal colon	7772 (76.3)	80 (52.3)	
Distal colon	2410 (23.7)	73 (47.7)	
pT stage			0.001
T1	315 (3.1)	0 (0.0)	
T2	1311 (12.9)	8 (5.2)	
T3	6536 (64.2)	104 (68.0)	
T4	2020 (19.8)	41 (26.8)	
pN stage			0.467
N0	6035 (59.3)	83 (54.2)	
N1a	1191 (11.7)	22 (14.4)	
N1b	1236 (12.1)	17 (11.1)	
N2a	854 (8.4)	13 (8.5)	
N2b	866 (8.5)	18 (11.8)	

Abbreviations: FJMUUH, Fujian Medical University Union Hospital; SEER, Surveillance, Epidemiology, and End Results.

### 3.2 LNR classification

**FIGURE 1:**
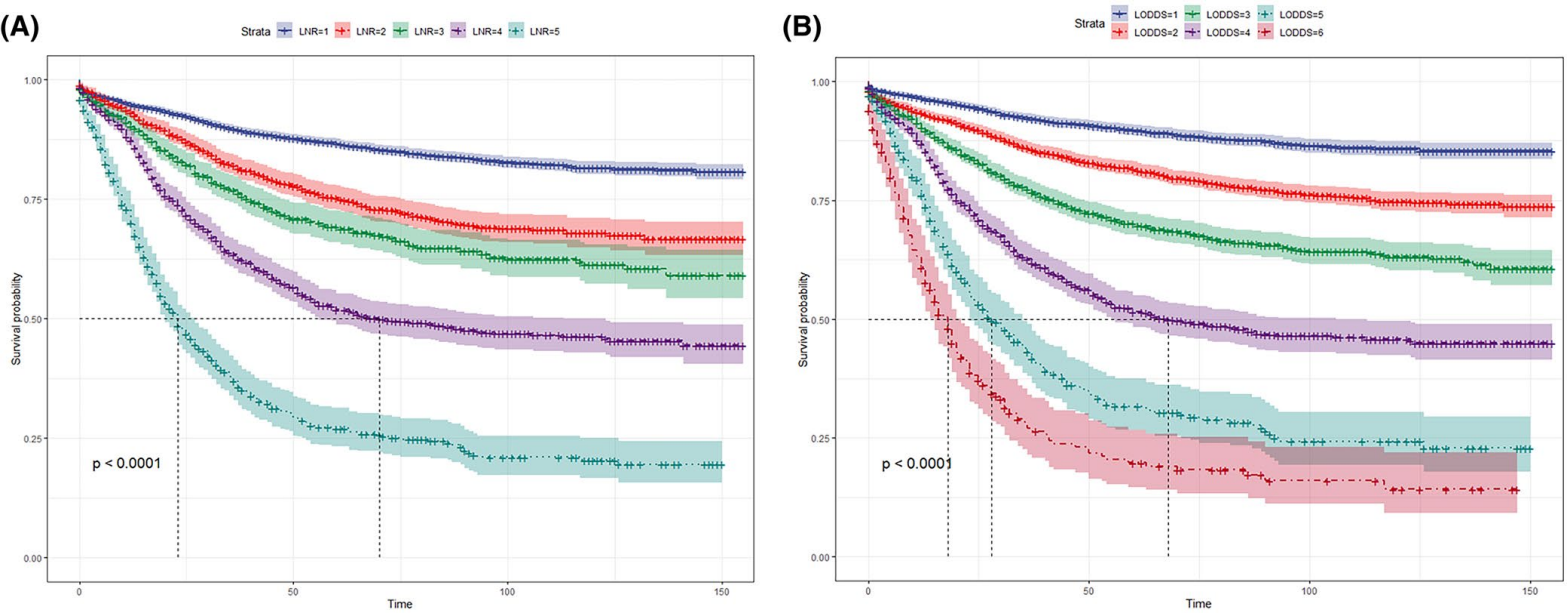
Kaplan–Meier survival curves for CSS in LNR classification (A) and LODDS classification (B). CSS, cancer‐specific survival; LNR, lymph nodes ratio; LODDS, log odds of positive lymph nodes

As shown in Table 2, we then compared the survival difference between LNR adjacent subgroup. The subgroup with similar prognoses was grouped together. Specifically, LNR classification: LNR1 (LNR = 0); LNR2 (0 < LNR ≤ 0.1); LNR3 (0.1 < LNR ≤ 0.2); LNR4 (0.2 < LNR ≤ 0.5); LNR5 (LNR > 0.5). Within this classification, 6035 (59.3%) in the LNR1 subgroup, 1456 (14.3%) in the LNR2 subgroup, 940 (9.2%) in the LNR3 subgroup, 1145 (11.2%) in the LNR4 subgroup, and 606 (6.0%) in the LNR5 subgroup. The 5‐year CSS was: 86.6%, 75.2%, 69.2%, 51.9%, 27.0% for the LNR1 subgroup to LNR5 subgroup, and the 8‐year CSS was 83.0%, 69.1%, 62.8%, 47.1%, 21.1%, respectively. KM plot presenting a significant difference in CSS among LNR classification as illustrated in Figure 1A (*p* < 0.001).

**TABLE 2:** 5‐year and 8‐year CSS in LNR subgroups

	*N* (%)	5‐year CSS (%)	8‐year CSS (%)	*p* value^a^
LNR = 0	6035 (59.3)	86.6	83.0	<0.001
0 < LNR ≤ 0.1	1456 (14.3)	75.2	69.1	0.001
0.1 < LNR ≤ 0.2	940 (9.2)	69.2	62.8	<0.001
0.2 < LNR ≤0.3	512 (5.0)	56.4	51.2	0.057
0.3 < LNR ≤ 0.4	375 (3.7)	50.5	44.9	0.517
0.4 < LNR ≤ 0.5	258 (2.5)	48.2	42.4	0.002
0.5 < LNR ≤ 0.6	153 (1.5)	34.6	27.1	0.572
0.6 < LNR ≤ 0.7	129 (1.3)	25.5	24.3	0.438
0.7 < LNR ≤ 0.8	110 (1.1)	29.8	16.3	0.091
0.8 < LNR ≤ 0.9	83 (0.8)	17.5	15.5	0.654
0.9 < LNR < 1.0	50 (0.5)	23.1	15.4	0.613
LNR = 1	81 (0.8)	24.0	19.4	

Abbreviations: CSS, cancer‐specific survival; LNR, lymph nodes ratio.

^a^ Comparison between adjacent subgroup groups.

### 3.3 LODDS classification

Next, we also grouped LODDS subgroups according to similar CSS. Detailed data are available in Table 3. Concretely, for LODDS classification: LODDS1 (LODDS ≤ −1.5); LODDS2 (−1.5 < LODDS ≤ −1.0); LODDS3 (−1.0 < LODDS ≤ −0.5); LODDS4 (−0.5 < LODDS ≤ 0); LODDS5 (0 < LODDS ≤ 0.5); and LODDS6 (LODDS > 0.5). Then, of this classification, 3510 (34.5%) in the LODDS1 subgroups, 3240 (31.8%) in the LODDS2 subgroups, 1748 (17.2%) in the LODDS3 subgroups, 1078 (10.6%) in the LODDS4 subgroups, 366 (3.6%) in the LODDS5 subgroups, and 240 (2.4%) in the LODDS6 subgroups. The 5‐year CSS was 89.7%, 81.6%, 70.1%, 51.5%, 31.7%, 19.8% for the LODDS1 subgroup to LODDS6 subgroup, and the 8‐year probabilities of CSS were 85.5%, 75.7%, 64.0%, 46.3%, 25.7%, 20.2%, respectively. Colon CSS was statistically different among LODDS classification (*p* < 0.001, Figure 1B).

**TABLE 3:** 5‐year and 8‐year CSS in LODDS subgroups

	*N* (%)	5‐year CSS (%)	8‐year CSS (%)	*p* value^a^
LODDS ≤ −2.0	84 (0.8)	90.0	90.0	0.617
−2 < LODDS ≤ −1.5	3426 (33.6)	89.7	86.6	<0.001
−1.5 < LODDS ≤ −1.0	3240 (31.8)	81.6	76.5	<0.001
−1.0 < LODDS ≤ −0.5	1748 (17.2)	70.1	64.7	<0.001
−0.5 < LODDS ≤ 0	1078 (10.6)	51.5	46.5	<0.001
0 < LODDS ≤ 0.5	366 (3.6)	31.7	24.3	<0.001
0.5 < LODDS ≤ 1	155 (1.5)	18.4	15.4	0.564
1 < LODDS ≤ 1.5	68 (0.7)	25.6	19.8	0.165
1.5 < LODDS ≤ 2	17 (0.2)	0.0	0.0	

Abbreviations: CSS, cancer‐specific survival; LODDS, log odds of positive lymph nodes.

^a^ Comparison between adjacent subgroup groups.

### 3.4 Comparison among three lymph nodes classifications

**FIGURE 2:**
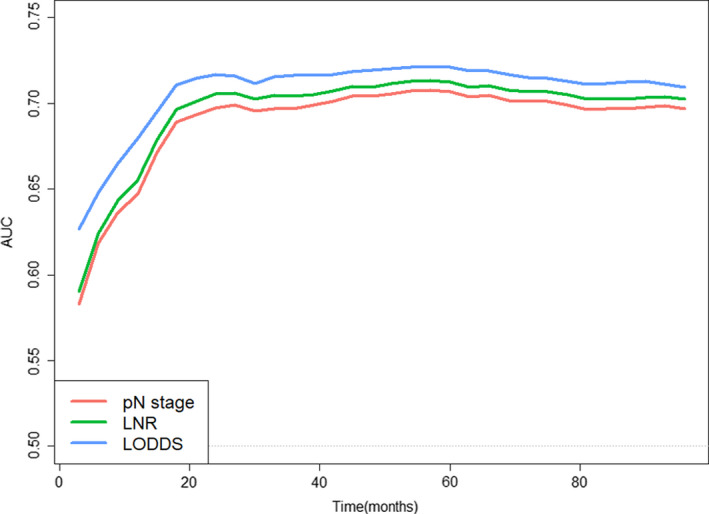
Time‐ROC used to compare CSS predictive capability of pN stage, LNR classification, and LODDS classification. AUC, the areas under the ROC curve; CSS, cancer‐specific survival; LNR, lymph nodes ratio; LODDS, log odds of positive lymph nodes; time‐ROC, time‐dependent receiver operating characteristic

To further demonstrate predictive capabilities of pN stage, LNR classifications, and LODDS classifications, time‐ROC were drawn, as shown in Figure 2. AUC of LODDS classifications was consistently outperformed pN stage and LNR classifications, which demonstrates the dominance of LODDS classifications in predicting CSS.

**FIGURE 3:**
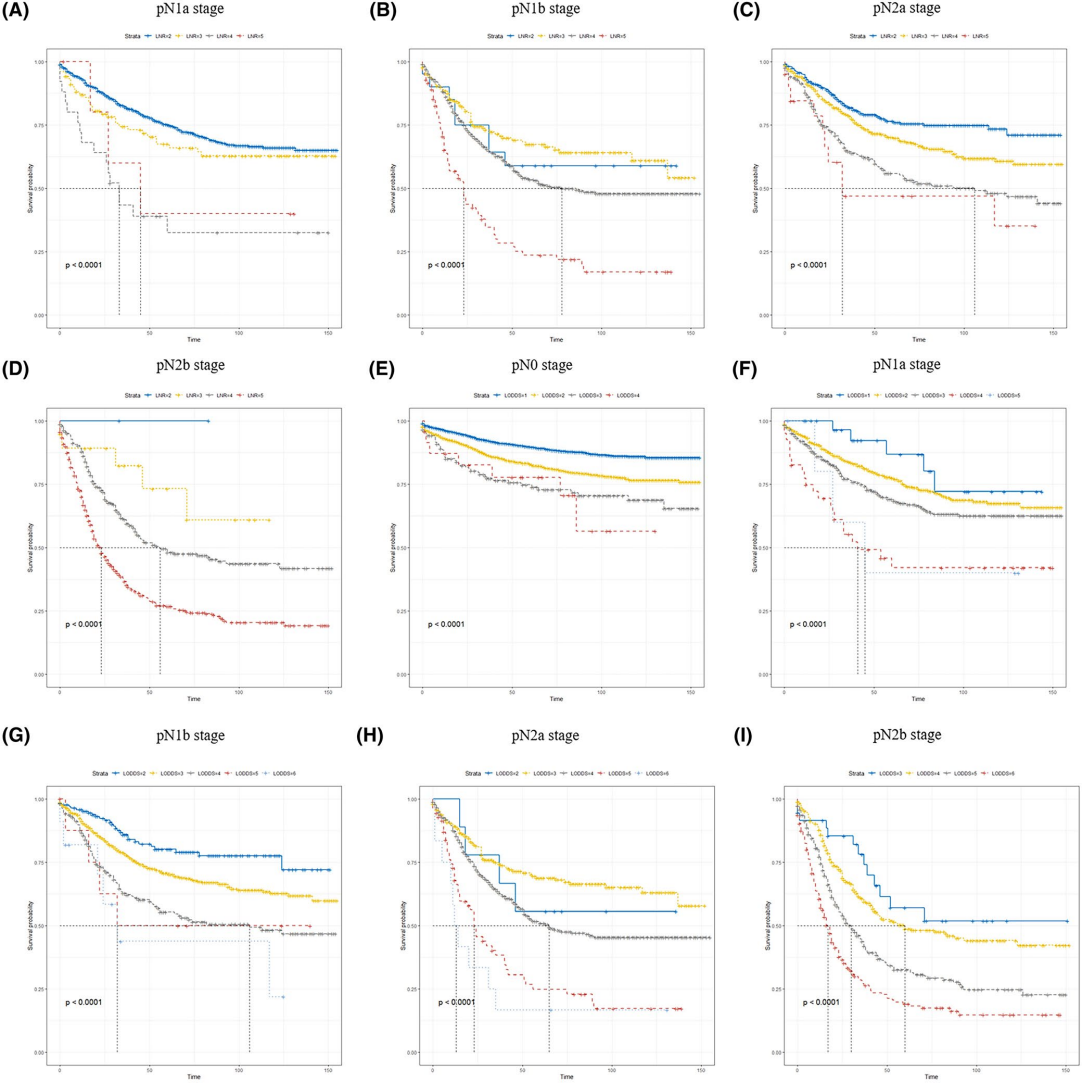
Kaplan–Meier survival curves for CSS in LNR (A–D) and LODDS (E‐I) classification according to pN stage. CSS, cancer‐specific survival; LNR, lymph nodes ratio; LODDS, log odds of positive lymph nodes

**FIGURE 4:**
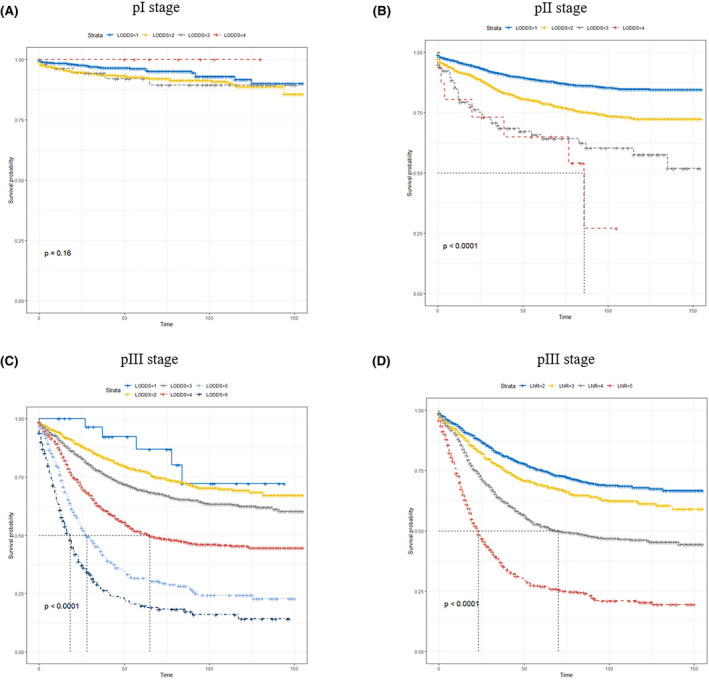
Kaplan–Meier survival curves for CSS in LODDS (A–C) and LNR (D) classification according to TNM stage. CSS, cancer‐specific survival; LNR, lymph nodes ratio; LODDS, log odds of positive lymph nodes; TNM, tumor‐node‐metastasis

Moreover, we contrasted CSS of patients based on three lymph nodes classifications. As is evident from Tables 4, 5 and 4, 5 and Figure 3, LNR and LODDS classifications can significantly distinguish the survival outcomes of patients with the same pN stage. However, this approach is not applicable for LNR classification in pN0 stage or LNR = 1. Patients with the same LNR and LODDS classifications presented a similar prognosis. Further, LODDS classification showed more remarkable prediction ability for the patient with LNR 1, 4, 5. As shown here (Figure 4), significant differences in CSS between LNR subgroups and LODDS subgroup were observed in TNM pIII stage, while this difference was not found for the LODDS subgroup in TNM pI stage.

**TABLE 4:** Comparison of CSS based on LNR classification

	LNR1		LNR2		LNR3		LNR4		LNR5		*p* value
	*N* (%)	8‐year CSS (%)	*N* (%)	8‐year CSS (%)	*N* (%)	8‐year CSS (%)	*N* (%)	8‐year CSS (%)	*N* (%)	8‐year CSS (%)	
pN stage											
pN0 stage	‐	‐	‐	‐	‐	‐	‐	‐	‐	‐	‐
pN1a stage	‐	‐	1058 (72.7)	67.1	102 (10.9)	62.7	25 (2.2)	32.5	6 (1.0)	40.0	<0.001
pN1b stage	‐	‐	376 (25.8)	74.9	606 (64.5)	62.4	234 (20.4)	50.1	20 (3.3)	46.9	<0.001
pN2a stage	‐	‐	20 (1.4)	58.9	213 (22.7)	64.1	539 (47.1)	48.5	82 (13.5)	17.0	<0.001
pN2b stage	‐	‐	2 (0.1)	100.0^a^	19 (2.0)	61.0	347 (30.3)	43.7	498 (82.2)	20.5	<0.001
*p* value	‐		0.256		0.887		0.131		0.292		
LODDS classifications											
LODDS1	3478 (57.6)	86.8	32 (2.2)	72.1	‐	‐	‐	‐	‐	‐	0.376
LODDS2	2360 (39.1)	78.7	880 (60.4)	70.3	‐	‐	‐	‐	‐	‐	0.001
LODDS3	173 (2.9)	70.5	544 (37.4)	66.8	925 (98.4)	62.8	106 (9.3)	59.7	‐	‐	0.446
LODDS4	24 (0.4)	56.5	‐	‐	15 (1.6)	59.1	1039 (90.7)	45.9	‐	‐	0.179
LODDS5	‐	‐	‐	‐	‐	‐	‐	‐	366 (60.4)	24.3	‐
LODDS6	‐	‐	‐	‐	‐	‐	‐	‐	240 (39.6)	16.0	‐
*p* value	<0.001		0.057		0.769		0.017		<0.001		

Abbreviations: CSS, cancer‐specific survival; LNR, lymph nodes ratio; LODDS, log odds of positive lymph nodes.

^a^ 5‐year CSS.

**TABLE 5:** Comparison of CSS based on LODDS classification

	LODDS1		LODDS2		LODDS3		LODDS4		LODDS5		LODDS6		*p* value
	*N* (%)	8‐year CSS (%)	*N* (%)	8‐year CSS (%)	*N* (%)	8‐year CSS (%)	*N* (%)	8‐year CSS (%)	*N* (%)	8‐year CSS (%)	*N* (%)	8‐year CSS (%)	
pN stage													
pN0 stage	3478 (99.1)	86.8	2360 (72.8)	78.7	173 (9.9)	70.5	24 (2.2)	56.5	‐	‐	‐	‐	<0.001
pN1a stage	32 (0.9)	72.1	702 (21.7)	68.7	411 (23.5)	63.2	40 (3.7)	42.0	6 (1.6)	40.0	‐	‐	<0.001
pN1b stage	‐	‐	169 (5.2)	77.5	841 (48.1)	65.2	206 (19.1)	50.5	9 (2.5)	50.0	11 (4.6)	43.8	<0.001
pN2a stage	‐	‐	9 (0.3)	55.6	287 (16.4)	66.4	476 (44.2)	45.4	70 (19.1)	17.2	12 (5.0)	16.7	<0.001
pN2b stage	‐	‐	‐	‐	36 (2.1)	51.8	332 (30.8)	44.1	281 (76.8)	24.7	217 (90.4)	14.8	<0.001
*p* value	0.376		0.002		0.681		0.371		0.307		0.458		
LNR classifications													
LNR1	3478 (99.1)	86.8	2360 (72.8)	78.7	173 (9.9)	70.5	24 (2.2)	56.5	‐	‐	‐	‐	<0.001
LNR2	32 (0.9)	72.1	880 (27.2)	70.3	544 (31.1)	66.8	‐	‐	‐	‐	‐	‐	0.057
LNR3	‐	‐	‐	‐	925 (52.9)	62.8	15 (1.4)	59.1	‐	‐	‐	‐	0.769
LNR4	‐	‐	‐	‐	106 (6.1)	59.7	1039 (96.4)	45.9	‐	‐	‐	‐	0.017
LNR5	‐	‐	‐	‐	‐	‐	‐	‐	366 (100.0)	24.3	240 (100.0)	16.0	<0.001
*p* value	0.376		0.001		0.446		0.179		‐		‐		

Abbreviations: CSS, cancer‐specific survival; LNR, lymph nodes ratio; LODDS, log odds of positive lymph nodes.

### 3.5 Prognostic factors of CSS

Univariate and multivariate Cox regression, as shown in Table 6, were performed to determine prognostic factors of CSS. On univariate analyses, colon CSS was significantly associated with age, tumor size, pT stage, pN stage, LNR, and LODDS (all *p* < 0.05). Next, pT stage, LNR, LODDS were individually included in three multivariate analyses. On multivariate analyses base on three independent lymph nodes classifications, there were significant correlations between CSS and pN stage (*p* < 0.001), LNR (*p* < 0.001), and LODDS (*p* < 0.001). Beyond this, age, tumor size, and pT stage were independent prognostic factors affecting CSS.

**TABLE 6:** Univariate and multivariable Cox analyses of CSS for colon MAC patients

Characteristics	Univariate			Multivariate 1			Multivariate 2			Multivariate 3		
	HR	95% CI	*p* value	HR	95% CI	*p* value	HR	95% CI	*p* value	HR	95% CI	*p* value
Age, years	1.021	1.017–1.024	<0.001	1.029	1.026–1.032	<0.001	1.029	1.025–1.032	<0.001	1.028	1.024–1.031	<0.001
Sex												
Male	Ref.			‐	‐	‐	‐	‐	‐	‐	‐	‐
Female	0.930	0.857–1.010	0.083	‐	‐	‐	‐	‐	‐	‐	‐	‐
Race			0.306									
White	Ref.			‐	‐	‐	‐	‐	‐	‐	‐	‐
Black	1.089	0.958–1.237	0.191	‐	‐	‐	‐	‐	‐	‐	‐	‐
Other/unknown	0.944	0.798–1.115	0.496	‐	‐	‐	‐	‐	‐	‐	‐	‐
Tumor size, cm												
≤5.5	Ref.			Ref.			Ref.			Ref.		
>5.5	1.275	1.175–1.384	<0.001	1.092	1.004–1.187	0.039	1.107	1.018–1.203	0.018	1.146	1.053–1.246	0.001
pT stage			<0.001			<0.001			<0.001			<0.001
T1	Ref.			Ref.			Ref.			Ref.		
T2	1.252	0.783–2.001	0.347	1.134	0.709–1.813	0.600	1.146	0.717–1.832	0.570	1.245	0.778–1.990	0.361
T3	3.428	2.228–5.275	<0.001	2.392	1.551–3.688	<0.001	2.427	1.574–3.742	<0.001	2.807	1.821–4.328	<0.001
T4	8.291	5.376–12.787	<0.001	5.274	3.407–8.165	<0.001	5.219	3.372–8.080	<0.001	6.056	3.914–9.371	<0.001
pN stage			<0.001			<0.001						
N0	Ref.			Ref.			‐	‐	‐	‐	‐	‐
N1a	2.069	1.813–2.360	<0.001	2.030	1.779–2.318	<0.001	‐	‐	‐	‐	‐	‐
N1b	2.415	2.133–2.734	<0.001	2.254	1.989–2.555	<0.001	‐	‐	‐	‐	‐	‐
N2a	3.791	3.342–4.301	<0.001	3.522	3.098–4.004	<0.001	‐	‐	‐	‐	‐	‐
N2b	6.254	5.584–7.003	<0.001	5.750	5.121–6.455	<0.001	‐	‐	‐	‐	‐	‐
LNR			<0.001						<0.001			
LNR1	Ref.			‐	‐	‐	Ref.			‐	‐	‐
LNR2	1.782	1.650–2.125	<0.001	‐	‐	‐	1.855	1.633–2.107	<0.001	‐	‐	‐
LNR3	2.429	2.116–2.787	<0.001	‐	‐	‐	2.246	1.955–2.579	<0.001	‐	‐	‐
LNR4	3.950	3.530–4.421	<0.001	‐	‐	‐	3.682	3.284–4.129	<0.001	‐	‐	‐
LNR5	8.292	7.346–9.360	<0.001	‐	‐	‐	7.262	6.414–8.221	<0.001	‐	‐	‐
LODDS			<0.001									<0.001
LODDS1	Ref.			‐	‐	‐	‐	‐	‐	Ref.		
LODDS2	1.891	1.657–2.159	<0.001	‐	‐	‐	‐	‐	‐	1.917	1.678–2.189	<0.001
LODDS3	3.102	2.704–3.559	<0.001	‐	‐	‐	‐	‐	‐	3.032	2.641–3.480	<0.001
LODDS4	5.384	4.687–6.184	<0.001	‐	‐	‐	‐	‐	‐	5.100	4.434–5.866	<0.001
LODDS5	9.561	8.089–11.301	<0.001	‐	‐	‐	‐	‐	‐	8.559	7.232–10.130	<0.001
LODDS6	14.214	11.820–17.094	<0.001	‐	‐	‐	‐	‐	‐	12.065	10.008–14.545	<0.001

Abbreviations: CI, confidence interval; CSS, cancer‐specific survival; HR, hazard ratio; LNR, lymph nodes ratio; LODDS, log odds of positive lymph nodes; MAC, mucinous adenocarcinoma.

### 3.6 Development and validation nomograms model

**FIGURE 5:**
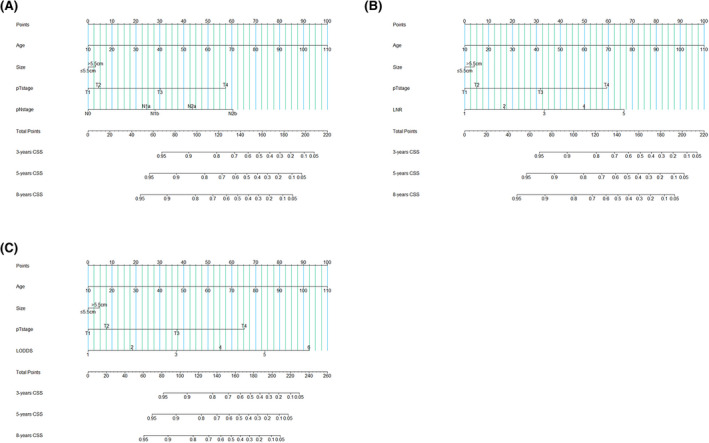
Nomograms based on pN stage (A), LNR classification (B), and LODDS classification (C) predicting CSS for colon MAC patients.CSS, cancer‐specific survival; LNR, lymph nodes ratio; LODDS, log odds of positive lymph nodes; MAC, mucinous adenocarcinoma

**FIGURE 6:**
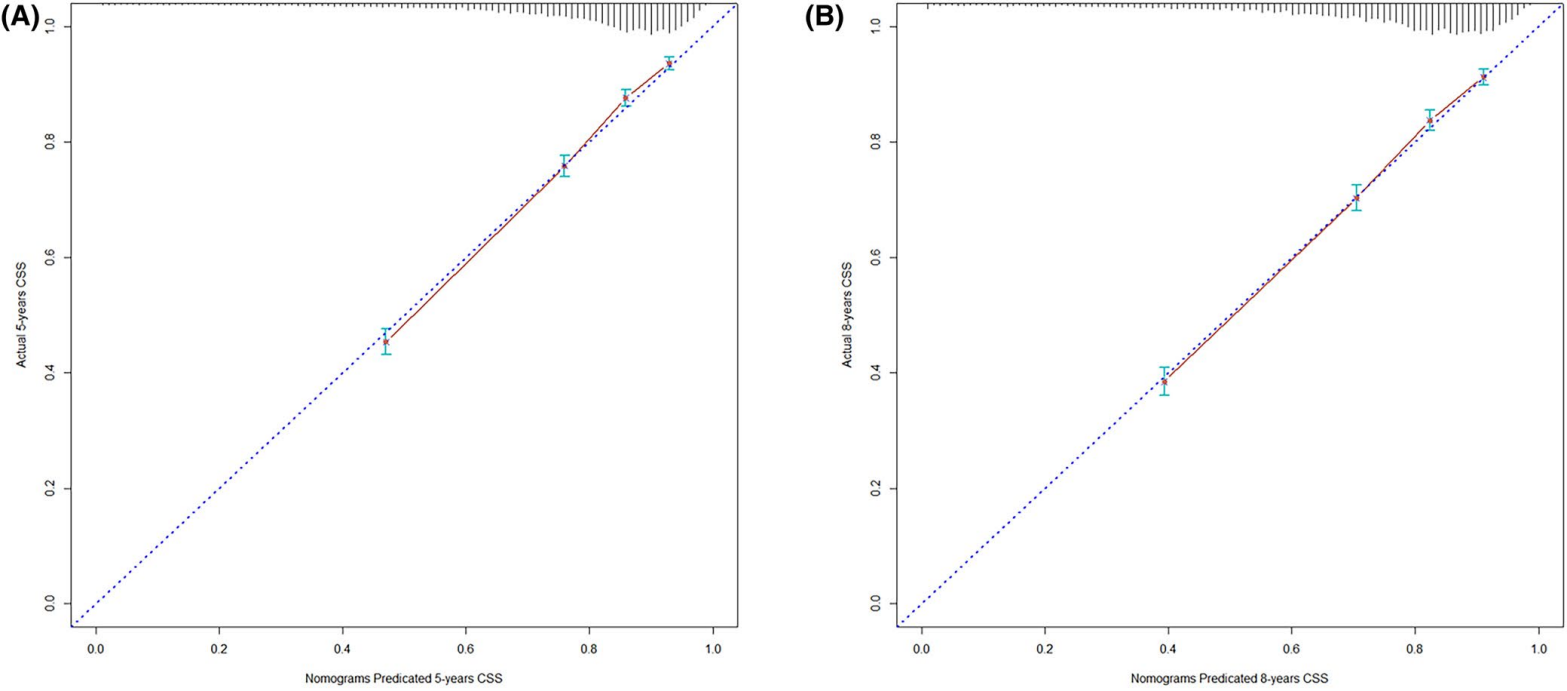
Calibration curve of 5‐year (A) and 8‐year (B) CSS for LODDS classification. CSS, cancer‐specific survival; LODDS, log odds of positive lymph nodes

Based on the multivariate analysis results, we constructed three nomograms at the base of three lymph nodes classifications, respectively, as shown in Figure 5. The C‐index of pN stage‐nomograms, LNR classification nomograms, and LODDS classification nomograms were 0.746 (95% confidence interval [95% CI]: 0.736–0.756), 0.750 (95% CI: 0.740–0.760), and 0.758 (95% CI: 0.748–0.768). The calibration plots predicting the 5‐year and 8‐year CSS also illustrated that LODDS classification nomograms are of great predictive capability (Figure 6).

**FIGURE 7:**
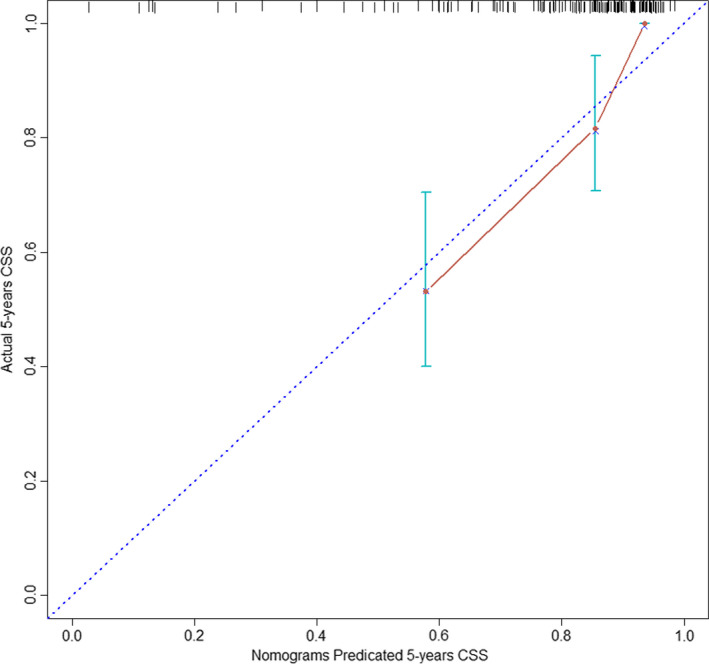
Calibration curve of 5‐year CSS in the external cohort. CSS, cancer‐specific survival

Furthermore, we validated LODDS classification nomograms using MAC patients from the FJMUUH cohort. The C‐index was 0.787 (95% CI: 0.648–0.926). The calibration curves predicting the 5‐year CSS, as shown in Figure 7, showed excellent agreement for nomograms.

### 3.7 Novel staging system based on recursive partitioning analysis

**FIGURE 8:**
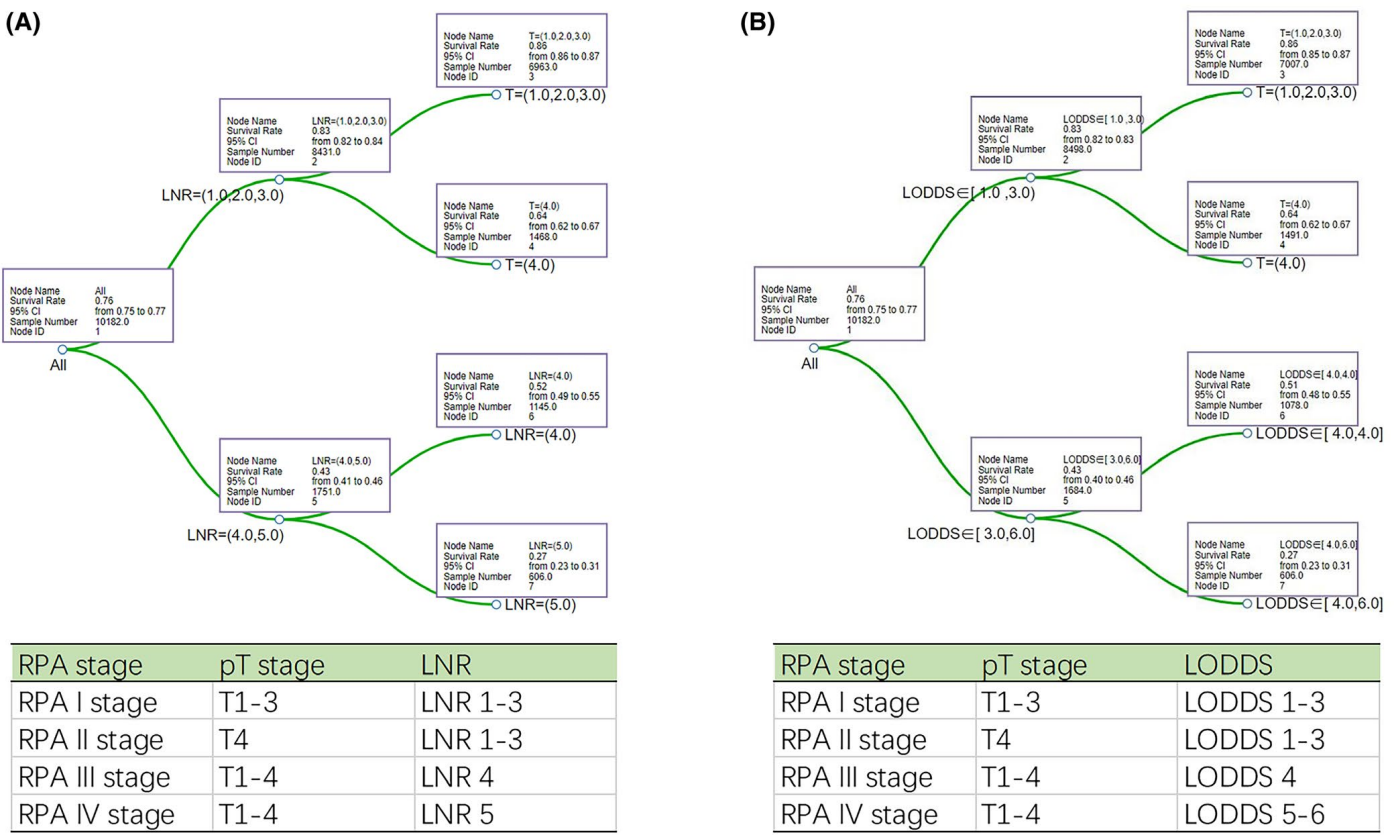
RPA stage based on LNR classification (A) and LODDS classification (B). LNR, lymph nodes ratio; LODDS, log odds of positive lymph nodes; RPA, recursive partitioning analysis

**FIGURE 9:**
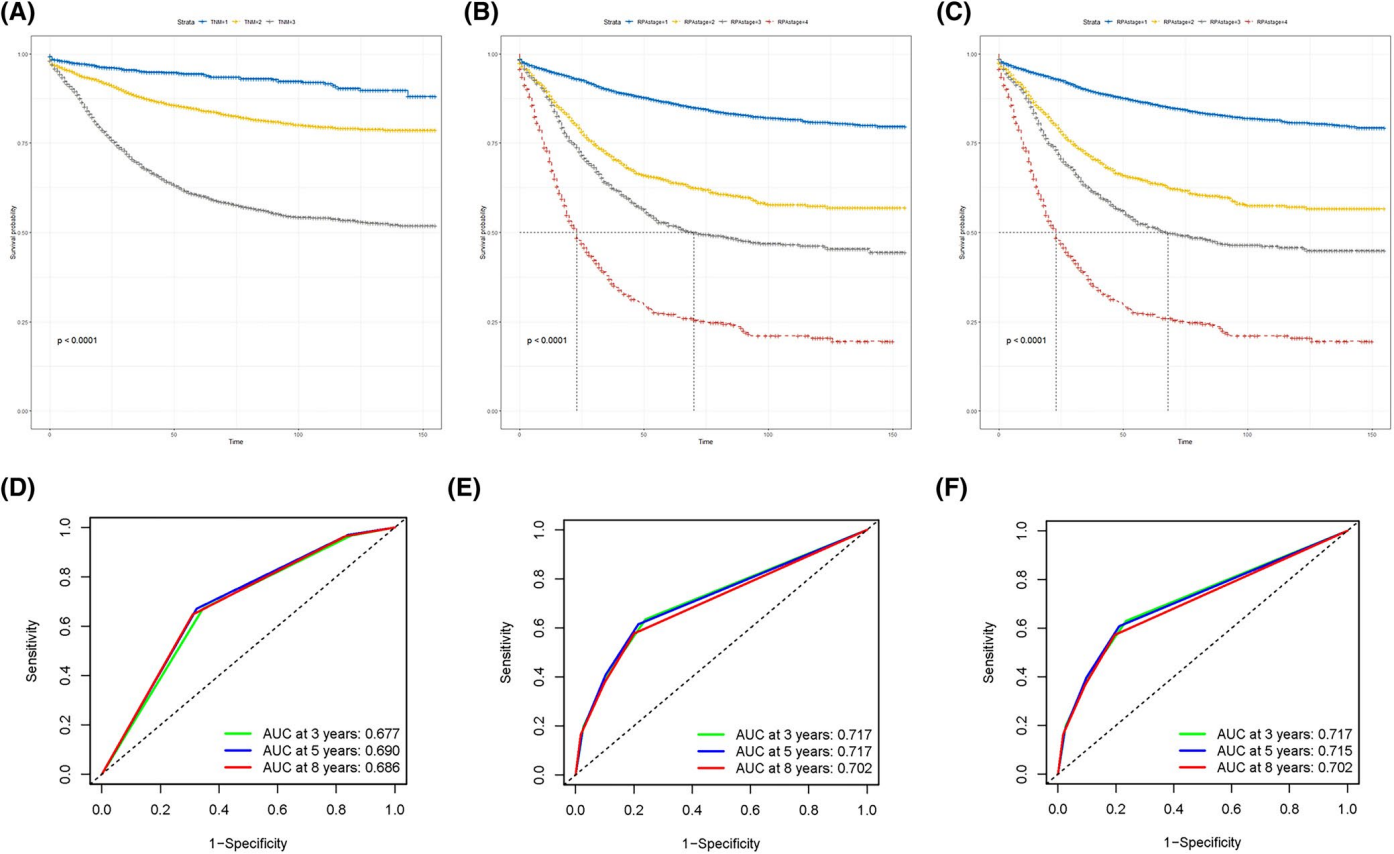
Kaplan–Meier survival curves and AUC for TNM stage (A, D), LNR‐RPA stage (B, E), and LODDS‐RPA stage (C, F). AUC, the areas under the ROC curve; LNR, lymph nodes ratio; LODDS, log odds of positive lymph nodes; RPA, recursive partitioning analysis

We develop a novel staging system using recursive partitioning analysis based on LNR classification (Figure 8A) and LODDS classification (Figure 8B), respectively. For LNR classification, RPA stage including RPA I stage (pT1–3, LNR1–3), RPA II stage (pT4, LNR1–3), RPA III stage (pT1‐4, LNR4), and RPA IV stage (pT1–4, LNR5). For LODDS classification, RPA stage including RPA I stage (pT1–3, LODDS1–3), RPA II stage (pT4, LOODS1–3), RPA III stage (pT1–4, LODDS4), and RPA IV stage (pT1–4, LODDS5–6). Further, a significant difference (*p* < 0.001) in CSS was found (Figure 9A–C) in TNM stage and the two RPA stages. The 3‐, 5‐, and 8‐year AUC of the LNR‐RPA stage and LODDS‐RPA stage were superior to TNM stage (Figure 9D–F).

## 4 DISCUSSION

In this study, we compared the predictive power of three lymph node classifications using a large population from the SEER database. LODDS showed great potential to distinguish colon MAC patients with differing clinical prognoses. This classification can distinguish different prognoses for patients with the same pN stage. As shown by time‐ROC, the predictive capability of LODDS classifications always outperforms pN stage and LNR classifications. Cox regression analysis revealed that three lymph node classifications (pN stage, LNR, LODDS) were significantly associated with CSS. Based upon the results, we constructed the nomograms prediction model, and have further confirmed the performance of this model using an external cohort. It can provide constructive information on prognosis to clinicians.

The histological type of cancer potentially correlates with biological properties and clinical outcomes. MAC is a relatively rare subtype that commonly manifests as an advanced stage and lymph node dissemination. Indeed, the effect of MAC on the survival of colon MAC patients remains debatable. Several studies have demonstrated that the MAC subtype was associated with a worse prognosis.^6^, ^9^, ^10^, ^11^, ^24^, ^25^, ^26^ In a population‐based survival study, MAC had a poor outcome because of advanced disease.^27^ This association has been confirmed in a large meta‐analysis.^28^ This study found that MAC portends worse survival. There was, however, a different result in other studies. They found that no differences between MAC and CA were observed in outcomes after correction for the AJCC stage.^12^, ^13^, ^14^, ^29^ Those findings reflected potential heterogeneity in the prognosis of MAC patients with the same pN stage. As one of the most dominant factors in affecting the survival outcomes, the lymph nodes status and lymph node staging can provide critical information. Thus, identifying and optimizing the prognosis risk factors and guidance personalized treatment and surveillance become important for colon MAC patients.

Lymph nodes ratio has proved to be the better approach to lymph nodes classification in a variety of cancers.^30^, ^31^, ^32^ A number of studies demonstrated LNR had prognostic value in patients with colon cancer.^33^, ^34^, ^35^, ^36^ Our findings in colon MAC patients were similar to previous studies. Specifically, in the present study, we observed that LNR classification could identify relatively good‐prognosis patients in the advanced pN stage (e.g., pN2a and pN2b stage) and poor‐prognosis patients in the early pN stage (e.g., pN1a stage).

The accurately discriminating prognosis was of significance in personalized treatment, and this approach enabled patients with advanced disease to have the confidence to active treatment. Whereas, unfortunately, the performance of LNR classification was limited for node‐negative patients.^37^ This limited the application and promotion of LNR classification.

In recent years, LODDS was introduced into cancer prognostic research. Benefiting from the unique computational approach, compared with LNR, LODDS largely circumvents the limitation of the status of negative lymph nodes and improves prognostic accuracy. In LODDS classification, the prognosis of those patients was further stratified by the number of negative lymph nodes, particularly in distinct differences in the number of retrieved. However, the outcomes of patients with negative lymph nodes were thought to be similar in LNR classification. On the other hand, our results revealed, there are differential outcomes between LODDS subgroups in the same LNR classifications. It implied LNR classification might be unable to discriminate well the survival of positive lymph nodes patients with the same LNR value but different total lymph node dissections. LODDS was significantly associated with overall survival as published in the study by Wang et al.^38^ However, their studies were limited to stage III colon cancer, while the role of LODDS in stage I/II colon cancer was not investigated.^19^, ^20^, ^21^ In addition, several previous studies were also suggested the value of LODDS was in prognosis prediction of non‐metastatic colon cancer. Indeed, this is a generalized observation for all histology types of colon cancer. Those results may not satisfy the need for personalized cancer therapies. Meanwhile, the lack of external validation limited the generalizability of those studies. In this context, we investigated in depth the potential role of LODDS in colon MAC patients and performed external validation using our cohort.

In the present study, we regroup MAC patients based on the value of LODDS with 0.5 intervals. Lastly, six subgroups were generated with significant differences in CSS between adjacent groups. In either pN stage, differences in survival among LODDS subgroups were observed. This implies that MAC patients with the same pN stage were heterogeneous, and LODDS classification can help clinicians identify different prognoses and develop personalized treatment and follow‐up strategies. Meanwhile, LODDS classification can aid patients who with lymph node metastases but the early LODDS subgroup enhances confidence in cancer therapy. However, for patients with pI stage, there were no differences in CSS in the LODDS subgroup, which was possibly due to early disease. Furthermore, Cox regression analysis LODDS was an independent risk factor of CSS. We further developed a nomograms model for three lymph nodes classifications, and LODDS classification nomograms exhibited the best performance toward prognostic stratification, compared with the AJCC stage and LNR. Meanwhile, we validated nomograms using an external validation cohort and demonstrated stable performance. Additionally, we constructed a new staging system based on pT stage and LODDS. In RPA stage, a better survival was observed in node‐positive MAC patients with early LODDS stage, which was clinically significant.

There are limitations in the present study. First, despite as a population‐based database, SEER lacked some tumor‐related (e.g., vascular invasion) and treatment‐related information (e.g., the details of radiotherapy and chemotherapy), and we cannot adjust potential confounders. Second, the inability to subdivide pT4a and pT4b status in the pT4 stage in patients diagnosed before 2010 from the SEER database, which made more accurate analyses difficult. Third, the number of external validation cohorts were relatively limited. As a consequence, subsequent studies should recruit a large sample and perform multicenter studies to further confirm and generalize our results.

## 5 CONCLUSION

In summary, we proved the superiority of LODDS in prognostic stratification for colon MAC patients compared with pN stage and LNR. LODDS classification nomograms and RPA stage can provide stable assessments of patient clinical outcomes and contributed to personalized cancer treatment.

## CONFLICT OF INTEREST

The authors have declared that no competing interests exist.

## Data Availability

The data of the SEER cohort were open access from the SEER database, and the data of the external validation group are available contacting the corresponding author.
